# Playing Hide and Seek: How Glycosylation of the Influenza Virus Hemagglutinin Can Modulate the Immune Response to Infection 

**DOI:** 10.3390/v6031294

**Published:** 2014-03-14

**Authors:** Michelle D. Tate, Emma R. Job, Yi-Mo Deng, Vithiagaran Gunalan, Sebastian Maurer-Stroh, Patrick C. Reading

**Affiliations:** 1 Centre for Innate Immunity and Infectious Diseases, Monash Institute of Medical Research, Monash University, Clayton, Victoria, 3168, Australia; E-Mail: michelle.tate@monash.edu; 2 Department of Microbiology and Immunology, The University of Melbourne, at the Peter Doherty Institute for Infection and Immunity, Victoria 3010, Australia; E-Mail: e.job@student.unimelb.edu.au; 3 WHO Collaborating Centre for Reference and Research on Influenza, Victorian Infectious Diseases Reference Laboratory, at the Peter Doherty Institute for Infection and Immunity, Victoria 3010, Australia; E-Mail: yi-mo.deng@influenzacentre.org; 4 Bioinformatics Institute, Agency for Science, Technology and Research, 138671, Singapore; E-Mails: vithiagarang@bii.a-star.edu.sg (V.G.); sebastianms@bii.a-star.edu.sg (S.M.-S.); 5 School of Biological Sciences, Nanyang Technological University, 639798, Singapore; 6 National Public Health Laboratory, Communicable Diseases Division, Ministry of Health, 169854, Singapore

**Keywords:** influenza virus, glycosylation, immune evasion, lectin

## Abstract

Seasonal influenza A viruses (IAV) originate from pandemic IAV and have undergone changes in antigenic structure, including addition of glycans to the hemagglutinin (HA) glycoprotein. The viral HA is the major target recognized by neutralizing antibodies and glycans have been proposed to shield antigenic sites on HA, thereby promoting virus survival in the face of widespread vaccination and/or infection. However, addition of glycans can also interfere with the receptor binding properties of HA and this must be compensated for by additional mutations, creating a fitness barrier to accumulation of glycosylation sites. In addition, glycans on HA are also recognized by phylogenetically ancient lectins of the innate immune system and the benefit provided by evasion of humoral immunity is balanced by attenuation of infection. Therefore, a fine balance must exist regarding the optimal pattern of HA glycosylation to offset competing pressures associated with recognition by innate defenses, evasion of humoral immunity and maintenance of virus fitness. In this review, we examine HA glycosylation patterns of IAV associated with pandemic and seasonal influenza and discuss recent advancements in our understanding of interactions between IAV glycans and components of innate and adaptive immunity.

## 1. Introduction

In the human population, influenza A viruses (IAV) are associated with acute respiratory illness and are responsible for millions of deaths annually. IAV express two membrane-bound surface glycoproteins, the hemagglutinin (HA) and the neuraminidase (NA), both of which express *N*-linked oligosaccharides. *N*-linked glycosylation is a common post-translational modification of mammalian glycoproteins whereby oligosaccharides (also referred to as carbohydrates, glycans, glycosites or saccharides) are attached through *N*-glycosidic linkages to the Asn residue of the glycosylation motif Asn-X-Ser/Thr-Y, where X/Y may represent any amino acid except proline [1]. *N*-linked glycosylation of the viral HA/NA occurs in the endoplasmic reticulum (ER) and Golgi apparatus in a process similar to that of host cell glycoproteins. The nature of the glycans expressed at different glycosylation sites on the HA/NA are determined by the particular host cell as well as the extent of intracellular processing [2,3]. HA and NA can contain a mixture of high-mannose (branched structures terminating in the sugar mannose), complex (branched structures terminating in galactose and/or *N*-acetyl-galactosamine (GalNAc)) or hybrid-type oligosaccharides [4,5]. *N*-linked glycans on influenza HA can be sulfated as a further structural modification [6,7]. Complex glycans expressed by the viral HA lack sialic acid (SIA) due to its removal by the action of the viral NA [8,9].

Sequence analysis can be used as a prediction tool but not as a definitive measure of the presence of glycans as potential glycosylation sites are not always occupied [10,11]. Such approaches rely on searching for the *N*-linked glycosylation sequence (Asn-X-Ser/Thr) in the amino acid sequence predicted by the viral RNA, however sequence is not the sole determinant of effective glycosylation nor does this provide any information regarding the composition of the specific glycans attached at occupied sites. For example, *N*-linked glycosylation is inhibited by particular combinations of Asn-X-Ser (e.g., Asn-Leu-Ser or Asn-Glu-Ser) or when the glycosylation motif is followed by specific amino acid sequences (e.g., Asn-X-Ser/Thr-Trp or Asn-X-Ser/Thr-Glu) [12,13]. Furthermore, potential sites in close structural proximity may not both be glycosylated due to steric hindrance and surrounding amino acids may also obstruct glycan attachment [11,14]. Clearly, biochemical approaches are required to determine glycan occupancy of potential sites as well as the composition of particular glycans attached to the HA.

## 2. IAVs Show Marked Differences in Glycosylation on the Head of the Viral HA

**Figure 1:**
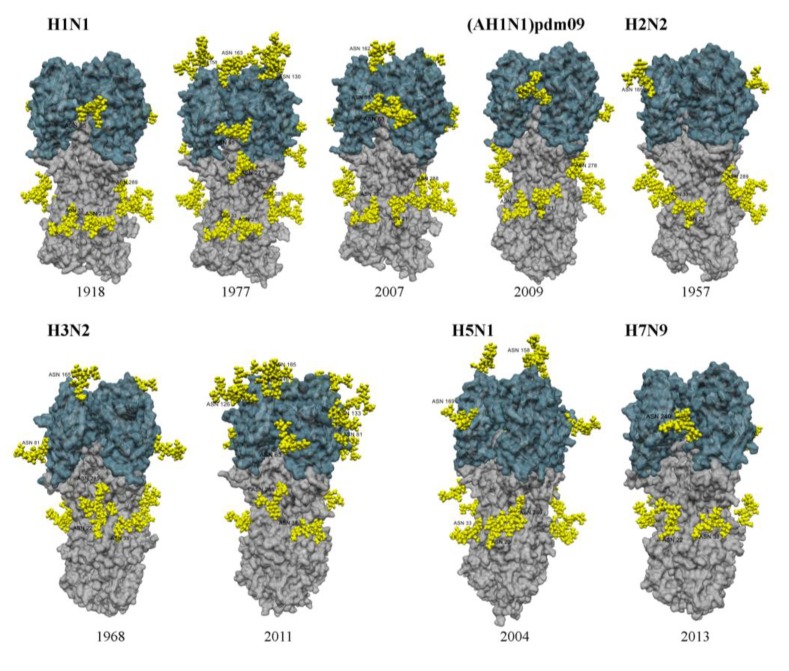
Structural models of the HA from different IAV subtypes showing glycosylation sites and attached glycans (yellow) on the head (blue) and stem (grey) of HA. HA proteins were derived from homology modeling based on representative strains from each subtype as indicated by * in Table 1. Glycosylation sites were derived from known glycosylated residues in closest known structures for each strain. Glycan molecules were manually added for each site using the Glyprot webserver [36] and energy minimisation was performed in Yasara (using the AMBER03 force field with default parameters) for glycans and adjacent HA atoms within 12Å as previously described [10]. Final models were rendered in POV-Ray [37]. Represenative HAs from H1N1, H2N2, H3N2, H5N1 and H7N9 viruses are shown.

Addition of glycans to the HA is thought to be an important mechanism contributing to antigenic drift and therefore sustained circulation of IAV in the human population [15,16]. Oligosaccharides attached to the stem/stalk region of the viral HA tend to be conserved across different virus strains, whereas those attached to the globular head display considerable variation in both number and location [15,17] (Table 1 and Figure 1). Note that for the purposes of this review H3 numbering has been used to indicate specific glycosylation sites and/or amino acid residues with the absolute HA numbering provided in parentheses for comparison (e.g., Asn_95_ (Asn_104_), or K133 (K147)). Glycans in the stalk region are critical for folding and conformation of the HA molecule [18,19,20,21] and removal of sites from the stalk resulted in impaired trimerization, folding and transport of HA to the cell surface [18,22], and altered the sensitivity of HA to changes in pH [21]. 

In the past century, influenza pandemics include the 1918 H1N1 ‘Spanish Flu’, the 1957 H2N2 ‘Asian Flu’, the 1968 H3N2 ‘Hong Kong Flu’ and the more recent 2009 A(H1N1)pdm09 ‘Swine Flu’. Other subtypes of avian and/or equine origin, including H5N1, H7N7, H7N9 and H9N2 have been implicated in sporadic but limited disease in humans; however these viruses have not yet evolved to transmit efficiently in the human population. Evolutionary studies of pandemic and seasonal H1N1 and H3N2 viruses indicate that the number of *N*-linked glycosylation sites on the head of HA increased after their emergence in the human population [14,23,24,25,26,27], suggesting that addition of glycans conferred a selective advantage, likely by preventing the binding of neutralizing antibody (Ab) to antigenic epitopes (see Section 4.1). In contrast, numbers of glycosylation sites on the N1 or N2 of human IAV are relatively stable, although the number does vary year to year [14,27]. Virus strains associated with the 1918 H1N1 pandemic expressed a single glycosylation site on the head of HA (Asn_95_ (Asn_104_)) [26,27], whereas H1N1 strains associated with subsequent epidemics (1930–1955) expressed 3–5 sites [26,27] and these remained relatively constant (1977–) until its disappearance in 2010 [14,26,27] (Table 1). Examples of glycosylation on representative H1 strains from 1918, 1977 and 2007 are shown in Figure 1. H3N2 strains associated with the ‘Hong Kong Flu’ pandemic of 1968 expressed 2 sites on the head of HA whereas recent strains expressed 6-7 sites [15,26] (Table 1 and Figure 1). The first cases of A(H1N1)pdm09 were identified in humans in April 2009 and had replaced seasonal H1N1 by 2010 [28]. Viruses associated with the A(H1N1)pdm09 pandemic also expressed only Asn_95_ (Asn_104_) on the head of the H1 HA [29,30] (Table 1 and Figure 1) and it has been proposed that A(H1N1)pdm09 may therefore evolve to acquire similar glycosylations to seasonal H1 HA [31,32]. However, A(H1N1)pdm09 glycosylation variants detected to date generally express Asn_123_ (Asn_136_) and/or Asn_165_ (Asn_179_) and these sites are distinct to those expressed by recent seasonal H1N1 viruses [10,29]. 

H2N2 strains associated with the ‘Asian Flu’ expressed a single overlapping glycosylation motif, which was highly conserved on the head of HA (residues 169–172 (NNTS)) and maintained throughout its circulation (1957–1968) in the human population [33,34] (Table 1 and Figure 1). Site‑directed mutagenesis demonstrated that either Asn_169_ or Asn_170_ (Asn_179_ or Asn_180_, respectively) can bear glycosylation, although their close proximity is likely to inhibit simultaneous expression of glycans [34]. Lack of glycan variability and/or the failure of the HA to acquire additional sites have been proposed as factors contributing to the relatively short circulation of H2N2 in man [25,26,34]. 

**Table 1:** Location and number of potential *N*-linked glycosylation sites on the hemagglutinin (HA) of different influenza A viruses (IAV). The glycosylation sites (Asn-Xaa-Ser/Thr) were predicted using NetNGlyc 1.0 server [35] which showed a threshold of above 0.5, then further confirmed to be present by structure modeling. Virus strains indicated by * were used to generate images in Figure 1.

Subtype	Virus	Stem		Head										Stem			GenBank/GISAID Acc No
**H3N2**	**H3 numbering**	22	38	63		81		126	133			165	246		276	285
		NGT	NAT	NCT		NET		NWT	NGT			NVT	NST		NCS	NGS
	A/Hong Kong/1/1968 *	+	+			+						+				+	CY044261
	**A/Bilthoven/1761/1976**	+	+	+				+				+				+	CY113197
	**A/Netherlands/620/1989**	+	+	+				+				+	+			+	CY113421
	**A/Shandong/9/1993**	+	+	+				+				+	+		+	+	CY108274
	**A/Panama/2007/1999**	+	+	+				+	+			+	+			+	CY112917
	**A/Brisbane/10/2007**	+	+	+				+	+			+	+			+	EPI353304
	A/Victoria/361/2011 *		+	+		+		+	+			+	+			+	EPI349103
**H1N1**	**H3 numbering**	21	33	63	65	95	123	129	130	158	162/163	165		271	278	288/289	
		NST	NVT	NCS	NIT	NGT	NTS	NHT	NTT	NGS	NLS	NNS		NAS	NTT	NSS	
	A/South Carolina/1/1918 *	+	+			+										+	AF117241
	**A/Wilson-Smith/1933**	+			+			+						+			DQ508905
	**A/Bellamy/1942**	+	+			+			+			+				+	HQ008263
	A/USSR/90/1977 *	+	+	+		+			+	+	+			+		+	DQ508897
	**A/Victoria/36/1988**	+	+			+		+			+					+	JX477163
	**A/New Caledonia/20/1999**	+	+	+		+		+			+					+	DQ508857
	A/Brisbane/59/2007 *	+	+	+		+					+					+	CY030230
																
	A/California/07/2009 *	+	+			+									+	+	EPI273609
	**A/Townsville/64/2010**	+	+			+	+								+	+	EPI294411
	**A/Perth/500/2010**	+	+			+						+			+	+	EPI269967
**H2N2**	**H3 numbering**	21	33									169	170			289	
		NST	NVT									NNT	NTS			NTT	
	A/Japan/305/1957 *	+	+									+				+	CY014976
	**A/Ann Arbor/7/1967**	+	+									+	+			+	CY125838
**H5N1**	**H3 numbering**	21	33							158		169		219		289	
		NST	NVT							NST		NNT		NRS		NSS	
	A/Vietnam/1194/2004 *	+	+							+		+				+	GQ149237
	**A/Cambodia/W0112303/2012**	+	+							+		+				+	JQ714246
	**A/Indonesia/5/2005**	+	+							+		+				+	CY116646
	**A/Turkey/12/2006**	+	+									+				+	EF619982
	**A/Egypt/321/2007**	+	+									+				+	EPI173707
	**A/Guizhou/1/2013**	+	+									+		+		+	EPI420386
**H7N9**	**H3 numbering**	22	38										240				
		NGT	NAT										NDT				
	A/Anhui/1/2013 *	+	+										+				EPI439507

Avian IAV of H1, H3, H5, H7 and H9 subtypes generally express fewer potential glycosylation sites of the head of HA compared to human seasonal IAV [38,39]. The H7N9 virus associated with human infections in China during 2013 is a reassortant virus expressing H7 and other gene segments from avian IAVs [40] and expresses a single glycosylation site on the head of its HA (Table 1 and Figure 1). Similarly, avian H5N1 strains generally express 1 or 2 sites on the head of HA (Table 1 and Figure 1). Analysis of 16 avian HA subtypes for sets of three codons that required single, double or triple nucleotide substitutions to produce potential glycosylation sequons found that H5 and H9 subtypes appear to have greater capacities to undergo mutations associated with HA glycosylation than past pandemic viruses [26]. 

## 3. HA Glycosylation Determines Sensitivity of IAV to Lectins of the Innate Immune System

**Figure 2:**
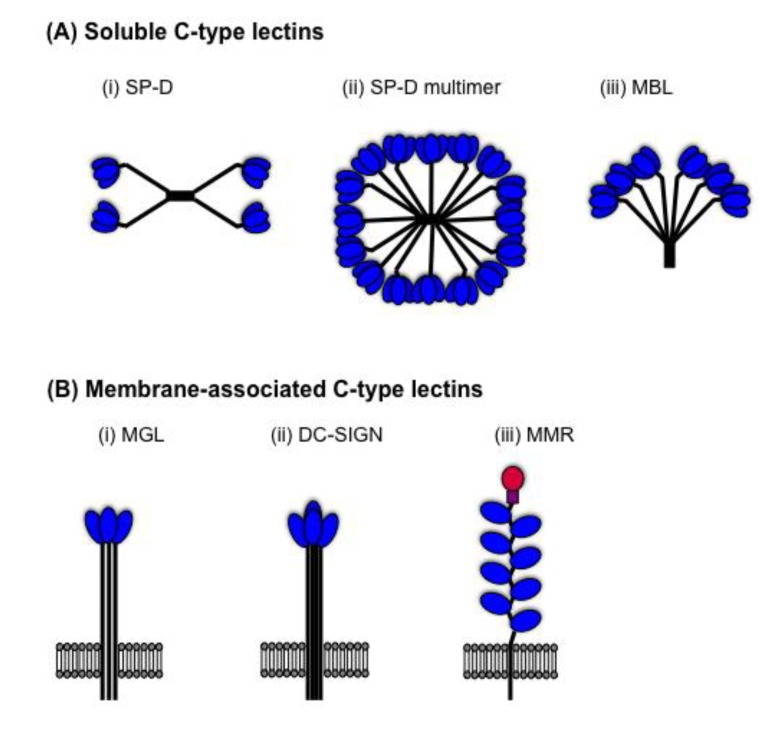
Structural organisation of mammalian C-type lectins. (**A**) Soluble C-type lectins of the collectin family are comprised of subunits containing three polypeptide chains (shown as black lines), each containing a single C-terminal carbohydrate recognition domains (CRD) (shown in blue). Trimeric subunits associate together to form (**i**) multimers with a characteristic cruciform-like structure (e.g., surfactant protein (SP-D)), or (**ii**) higher-order multimers (e.g., SP-D), or (**iii**) multimers with a bouquet-like structure (e.g., mannose-binding lectin (MBL)). (**B**) Membrane-associated C-type lectins. The domain organization of the type II transmembrane proteins (**i**) macrophage galactose-type lectin (MGL) and (**ii**) DC-specific intercellular adhesion molecule-3-grabbing non-integrin (DC-SIGN) show a polypeptide chain containing a single CRD (shown in blue) which cluster together to form homo-oligomers on the cell surface. (**iii**) The macrophage mannose receptor (MMR) is a type I transmembrane protein which contains 8 CRDs (shown in blue) on a single polypeptide chain, as well as a cysteine-rich domain (red circle) and a fibronectin domain (purple square).

Innate host defences have evolved to detect *N*-linked glycans present on the surface of microbial pathogens, including IAV. Innate inhibitors of IAV are soluble proteins in serum and respiratory secretions that mediate a range of anti-IAV activities against virions and virus-infected cells (reviewed in [41,42]). In 1990, Anders *et al.* reported that β inhibitors in mammalian serum were Ca^2+^-dependent (C-type) lectins that bound to mannose-rich glycans on IAV HA to neutralize virus infectivity [43,44]. Since this time, the anti-IAV activities of soluble C-type lectins of the collectin family, such as mannose-binding lectin (MBL) and surfactant protein (SP)-D, have been widely reported. In addition, membrane-associated C-type lectins on macrophages (Mφ) and dendritic cells (DC), such as the macrophage mannose receptor (MMR), macrophage galactose-type lectin (MGL) and DC-specific intercellular adhesion molecule-3-grabbing non-integrin (DC-SIGN), have been implicated in innate immunity to IAV. Soluble and membrane-associated C-type lectins contain one or more highly conserved carbohydrate recognition domains (CRDs) which allow recognition of a broad range mannose/*N*-acetyl-glucosamine-type (Man-type) structures or galactose/*N*-acetyl-galactosamine-type (Gal-type) sugars (reviewed in [45]), which are common on the surface of microbial pathogens but rarely expressed on endogenous glycoproteins. Collectin subunits are comprised of 3 identical or similar polypeptide chains associated together to form triple helices. Subunits may associate together to form multimers with characteristic ‘cruciform-like’ (e.g., SP-D, Figure 2A(i)) or ‘bouquet-like’ structures (e.g., MBL, Figure 2A(iii)), or higher order oligomers (see Figure 2A(ii)), thereby increasing avidity for microbes and other ligands. Membrane-associated C-type lectins expressing a single CRD (e.g., MGL, DC-SIGN) form homo-oligomers at the cell-surface to increase binding avidity (Figure 2B(i)/(ii)). In contrast, the MMR contains multiple CRDs on a single polypeptide chain (Figure 2B(iii)).

### 3.1. Soluble C-Type Lectins

SP-D and MBL bind to oligosaccharides on the viral HA/NA to mediate a range of anti-IAV activities, including hemagglutination inhibition, neutralization of virus infectivity, viral aggregation, inhibition of the enzymatic activity of the viral NA and protection of neutrophils against IAV-induced neutrophil dysfunction (reviewed in [42,46]). SP-D is constitutively expressed in the airways and levels increase during IAV infection of mice [47]. Importantly, SP-D has been shown to contribute to the neutralizing activity of bronchoalveolar lavage fluids (BALF) from humans and mice against highly glycosylated IAV [48,49,50,51]. MBL is produced in the liver although it has been detected in human airway fluids during inflammation [52] and in BALF from IAV-infected mice [47]. Studies using knockout mice have clearly demonstrated roles for SP-D [53,54,55,56] and MBL [57] in innate immune defence against IAV infection and highlight the importance of SP-D in particular against highly glycosylated IAV [53,54,55]. Other members of the collectin family, including conglutinin, CL kidney 1 (CL-K1, also known as CL-11), CL-43 and CL-46 also mediate anti-IAV activity *in vitro* [44,58,59,60,61]. 

Reverse genetic approaches have been widely utilized to examine the effect of addition or removal of potential glycosylation sites from the HA in the context of a virus ‘backbone’ that is genetically identical. Addition of sites to the head of A/Hong Kong/1/68 (H3N2) HA resulted in increased sensitivity to SP-D *in vitro* and attenuated virulence in mice [55] whereas removal from the globular head of H3 (A/Beijing/353/89) and H1 (A/Brazil/11/78) HA_1_ led to resistance to neutralization by SP‑D and increased virulence in mice [62,63]. Of interest, addition of glycans to the HA of the mouse‑adapted PR8 virus, which lacks glycans on the head of its HA [64], resulted in sensitivity to SP-D and attenuated virulence in mice [62]. These genetically defined viruses confirmed the importance of particular glycosylation sites (e.g., Asn_165_ (Asn_181_) for H3 and Asn_130_ (Asn_144_) for H1) in determining sensitivity to rodent [62,63] and human SP-D [29]. Recently, similar approaches confirmed that addition (to virus expressing the 1918 pandemic H1 HA) or removal (to virus expressing the H1 HA of seasonal A/Solomon Islands/2006) of glycosylation sites from the head of HA was associated with attenuated or enhanced virulence in mice, respectively [65], although sensitivity to SP-D was not addressed in this study.

It is clear that the degree of glycosylation on the globular head of H1 and H3 seasonal IAV is a critical determinant of sensitivity to SP-D and MBL from humans or mice [47,66]. Consistent with these findings, IAV strains associated with pandemics or from zoonotic sources (including some with pandemic potential) generally express low levels of HA glycosylation (see Figure 1 for 1918 H1, 1968 H3, A(H1N1)pdm09, H5 and H7) and are therefore resistant to lectin-mediated inhibition by collectins. For example, A(H1N1)pdm09 express a single potential glycosylation site on the globular head of HA and a number of studies confirmed these viruses to be resistant to SP-D and MBL [10,29,67]. Chimeric IAV expressing the HA of 1918 (H1, 1 site), 1957 (H2, 2 sites), 1968 (H3, 2 sites) or 2009 (H1, 1 site) pandemic viruses were all largely resistant to SP-D and induced significant lung pathology in mice whereas a virus expressing the HA of a seasonal IAV induced mild lung pathology and was sensitive to SP-D [68]. Data reported to date indicate that H5N1 [66] and recent H7N9 IAV from China [69] are also resistant to SP-D.

### 3.2. Membrane-Associated C-Type Lectins

Infection of Mφ and DCs by seasonal IAV is generally abortive [48,70,71,72,73,74,75], but does result in release of anti-viral and pro-inflammatory cytokines [48,72], which may control early virus replication and regulate inflammatory responses to infection. Depletion of airway Mφ and/or particular DC populations has been associated with enhanced IAV replication and exacerbated disease in mice [76,77,78,79,80], arguing that infection of Mφ/DC may be an important factor limiting the severity of IAV-induced disease. While interactions between the viral HA and cell-surface SIA clearly modulate the susceptibility of Mφ to IAV infection [62,63,76], recent studies suggest that C-type lectins, including MMR, MGL and DC-SIGN, act as attachment and/or entry receptors for IAV infection of Mφ and DC (reviewed in [81]).

Early studies from our group demonstrated that IAV expressing a highly glycosylated HA (e.g., BJx109) infected murine Mφ to high levels whereas poorly glycosylated strains (e.g., PR8) did not, while both viruses infected epithelial cells to equivalent levels [82]. Moreover, IAV infection of Mφ, but not epithelial cells, was inhibited by mannan [82] and depletion of airway MΦ from the lungs of mice prior to infection with the avirulent BJx109 strain resulted in severe disease [76]. Subsequent biochemical approaches demonstrated Ca^2+^-dependent, lectin-mediated interactions between IAV and both MMR and MGL [82,83]. Recently, we confirmed that expression of murine MGL1 in IAV‑resistant Lec1 CHO cells restored susceptibility to infection [84]. Moreover, Lec1 cells expressing endocytosis-deficient MGL1 bound IAV efficiently, but were largely resistant to infection, indicating that direct internalization via MGL1 can result in cellular infection. 

DC-SIGN functions as an attachment and/or entry receptor for a range of viruses expressing glycosylated surface antigens (reviewed in [81]) and recent studies indicate it also modulates IAV infection of target cells. Wang *et al.* implicated DC-SIGN as an attachment factor for H5N1 IAV resulting in enhanced virus infection *in* *trans* as well as promoting virus entry *in cis* via additional interactions with sialylated cell-surface molecules [85]. Subsequent studies from our group demonstrated that SIA-deficient CHO Lec2 were resistant to IAV infection, however Lec2 cells expressing DC-SIGN bound IAV in a Ca^2+^-dependent manner and were susceptible to infection [86]. Moreover, viruses bearing a highly glycosylated HA (e.g., BJx109) infected Lec2-DC-SIGN cells to high levels whereas the poorly glycosylated PR8 strain did not. Recently, Hillaire *et al.* confirmed the importance of HA glycosylation in determining susceptibility to DC-SIGN-mediated infection and implicated the receptor in SIA-independent infection of human DC [87]. Glycan-binding receptors have also been implicated in attachment and infection of chicken DC by avian IAV, although the specific identity and role of these receptors has not yet been defined [88].

## 4. HA Glycosylation and Immune Evasion by IAV

### 4.1. HA Glycosylation Alters Ab-Mediated Recognition of Antigenic Epitopes

It has long been recognized that acquisition of *N*-glycans on the globular head of the HA can mask or modify antigenic sites recognized by neutralizing Abs. Studies in the early 1980s describing the three-dimensional structure of the 1968 H3 HA noted that oligosaccharide attachment sites were present in regions implicated in Ab binding [89] and similar observations were subsequently recorded for H1 HA [64,90]. In 1984, Skehel *et al.* demonstrated that addition of a glycosylation site at residue 63 of HA_1_ blocked binding of a mAb to H3 HA, providing experimental evidence of carbohydrate-mediated modification of HA antigenicity [91]. Subsequent studies confirmed that addition of new oligosaccharides to the head of HA could reduce reactivity with mAbs and/or polyclonal antisera [15,31,32,33,92]. mAbs targeting highly conserved epitopes in the stem region of HA can neutralize most H1 IAV, as well as H2 and H5 subtype viruses [93,94], however binding to H3 appears to be blocked by an *N*-linked glycan which is not expressed by H1/H2/H5 HAs [95].

Compared to seasonal IAV, A(H1N1)pdm09 caused greater morbidity and mortality in children and young adults [96]. In a number of studies, the elderly (>65 years old) showed a higher prevalence of cross-reactive Abs [97,98,99], suggesting that they were previously exposed to a virus or vaccine that was antigenically related. Investigations to understand why severe disease and hospitalization associated with A(H1N1)pdm09 predominated in younger age groups renewed interest and led to a deeper understanding of the role HA glycosylation in antigenic masking and protection. O’Donnel *et al.* demonstrated that infection of ferrets with H1N1 IAV isolated in and prior to 1947 provided significant protection against subsequent challenge with A(H1N1)pdm09, however antigenic changes in H1 occurred between 1947–1950, such that prior infection no longer induced cross-protection [100]. Loss of antigenic cross-reactivity was associated with addition of glycosylation sites to H1, leading the authors to propose this as a likely explanation for the observation that the elderly were protected against A(H1N1)pdm09 infection while younger adults were more susceptible [100]. Wei *et al.* investigated the basis of antibody-mediated cross-neutralization between A(H1N1)pdm09 and 1918 H1N1 pandemic viruses, demonstrating that a conserved site at the sub-region of the HA receptor binding domain served as a major target for cross-neutralizing antibodies. However, some seasonal H1N1 strains ‘masked’ this site by addition of two glycosylation sites—Asn_129_ (Asn_142_) and Asn_163_ (Asn_177_) and addition of these sites to A(H1N1)pdm09 or 1918 H1N1 pandemic viruses rendered them resistant to antisera raised against either wild-type virus [31]. 

Studies described above have provided important insights regarding the ability of HA glycosylation to mask antigenic sites and to modulate antigenic properties of H1 HA, however it is important to note that A(H1N1)pdm09 variants expressing Asn_142_, Asn_144_, and Asn_177_ (Asn_129_, Asn_130_ and Asn_163_) have not been reported to date. Instead, variants express Asn_123_ and/or Asn_165_ (Asn_136_ and/or Asn_179_) [10,29,101] and addition of Asn_123_ (Asn_136_) to the head of A(H1N1)pdm09 HA was associated with resistance to neutralizing antibodies raised in mice or in ferrets [10]. Moreover, variants expressing Asn_123 _(Asn_136_) displayed enhanced ‘breakthrough’ and growth in the airways of mice that received monovalent A(H1N1)pdm09 vaccine, suggesting evasion of antibody-mediated immunity *in vivo.*

Masking antigenic sites on the globular head of HA with glycans may have important implications regarding the focus and/or the magnitude of humoral response elicited. For example, infection of mice with a glycosylated virus (expressing the HA of pandemic 1968 H3N2 strain with 4 additional glycosylation sites added) elicited poor neutralizing antibody responses and did not protect mice from re-infection with a poorly glycosylated variant (expressing the wild-type HA of 1968 H3N2) [102]. A similar scenario was observed when mice were infected sequentially with a glycosylated seasonal H1N1 virus, followed by a poorly glycosylated A(H1N1)pdm09 strain. Moreover, mice re-infected with poorly glycosylated variants developed severe disease, characterized by robust T cell-mediated immunopathology leading the authors to propose that the disproportionate incidence of severe disease sometimes observed in young adults during pandemic influenza may be mediated, in part, by robust T cell responses as a result of previous influenza infections in the setting of inadequate antibody neutralization [102]. 

Recent studies clearly demonstrate that HA glycosylation can modulate the induction of cross-reactive antibody responses and/or focus humoral immunity to different regions of HA. For example, addition of Asn_130_ (Asn_144_), a site expressed by many seasonal H1N1 strains between 1930–1938, to A(H1N1)pdm09 HA was shown to shield an immunodominant region of HA, likely the Sa site [32] however A(H1N1)pdm09 virus bearing Asn_130_ (Asn_144_) elicited a broader polyclonal response, which appeared to be against multiple antigenic regions in the HA and cross-neutralized a panel of wild-type and glycosylation mutant viruses [32]. Eggink *et al.* hyper-glycosylated recombinant HA in an attempt to shield immunodominant epitopes on the globular head and redirect antibody responses towards the conserved stalk domain of HA [103]. Immunization with hyper-glycosylated HA induced higher titres of antibodies directed to the HA stalk while dampening the immune response to the globular head domain. Moreover, mice immunized with hyper-glycosylated HA were better protected following challenge with a virus expressing an irrelevant head domain but a shared stalk domain of HA, demonstrating the importance of HA stalk-directed immunity *in vivo* [103]. 

Recombinant HA proteins differ in their glycosylation state as a result of different protein expression systems and/or processing conditions and this, in turn, can influence their ability to induce humoral responses. For example, HAs expressing terminal mannose moieties, such as those produced in insect cells, induced lower antibody titers than those produced in mammalian cells which expressed complex glycans or single *N*-acetyl glucosamine (GlcNAc) moieties [104]. In another study, antibodies elicited to H5 HA bearing only a single *N*-linked GlcNAc at each glycosylation site were shown to mediate better neutralizing activity and enhanced protection following challenge with a lethal dose of H5N1 compared to fully glycosylated HA [105]. HA amino acid sequences around glycosylation sites generally show less variation [25] and therefore expression of smaller glycans may result in immunogens that induce broader immunoreactivity compared to their fully glycosylated counterparts. Clearly, glycosylation state will be an important factor impacting on the development of recombinant HA-based vaccines for use against IAV.

### 4.2. Effects of HA Glycosylation on T Cell-Mediated Immunity to IAV

Conventional trivalent influenza vaccines (inactivated split virus) induce humoral immunity whereas both antibody and T cell responses are generated following natural IAV infection. T lymphocytes are largely directed towards conserved epitopes of internal proteins such as PA and NP, and therefore can provide cross-protection between different IAV subtypes (reviewed in [106]). However, CD4^+^ and CD8^+^ T cell epitopes derived from the HA protein have also been described [107]. While the ability of naturally occurring variants of IAV to escape T cell recognition as a consequence of changes in HA glycosylation has been proposed [108,109,110], there is limited experimental evidence describing modulation of T cell responses to IAV by HA glycosylation. Using CD4^+^ T cell clones, Drummer *et al.* reported that partial deglycosylation of HA exposed stimulatory determinants and that complete deglycosylation resulted in functional loss of some T cell determinants but not others [111]. Furthermore, addition of carbohydrates to HA peptides interfered with CD4^+^ T cell responses *in vitro*, leading the authors to propose that glycans inhibited the approach of clones bearing certain T cell receptors to the glycopeptide-MHC complex [112]. 

## 5. Impact of HA Glycosylation on IAV Biology

### 5.1. Impact of HA Glycosylation on HA Receptor Avidity and Virus Fitness

It is well established that oligosaccharides in close proximity to the receptor-binding site (RBS) of HA can also alter its binding avidity and/or specificity for sialylated receptors. Generation of mAb escape mutants indicate that H2 can acquire at least one new glycosylation site at one of three distinct locations on the HA, however impaired receptor-binding and cell-fusion activities suggest that glycan addition comes with a significant cost to virus fitness [33,34] and may explain why glycosylation variants did not emerge and circulate in humans. Oligosaccharides expressed by H1 [65,92,113,114,115], H3 [15,43,63,116], H5 [117,118] and H7 HA [119] have all been shown to impact HA receptor specificity, providing evidence that the advantages associated with glycan-mediated evasion of humoral immunity may be offset by defects in the biological activities of HA.

Recently, Das *et al.* [120] investigated the effects of HA glycosylation on receptor avidity and virus fitness in an effort explain the paucity of oligosaccharides on HA compared to other viral receptor proteins such as HIV gp160 [121,122]. Escape mutants of PR8 selected in the presence of an anti-HA mAb expressed an additional *N*-glycosylation site on the viral HA at Asn_121_ or Asn_130_ (Asn_131_ or Asn_144_, respectively) but were always characterized by compensatory mutations (both in the HA and/or NA) to mitigate reduced receptor avidity. Sequence analysis of circulating H1 IAV confirmed that natural occurrence of glycosylation at Asn_121_ (Asn_131_) was always accompanied by a compensatory mutation known to increase receptor avidity whereas Asn_130_ (Asn_144_) was not detected amongst circulating H1 IAV [120]. Studies from our group have also demonstrated that addition of Asn_123 _(Asn_136_) to the HA of A(H1N1)pdm09 altered receptor specificity and that infection of naïve or vaccinated mice with mutants bearing this glycan selected for compensatory mutations in the HA known to alter receptor avidity [10]. Kim *et al.* recently demonstrated the importance of polymorphism in H1 HA at residue 133 (147) in compensating for the loss of virus replication, virulence and transmissibility associated with addition of glycosites at Asn_129_ and Asn_163_ (Asn_142_ or Asn_177_, respectively), and proposed that K133 (K147) may protect the receptor-binding pocket from steric hindrance associated with glycosylation at these residues [101]. Thus, HA glycosylation can interfere with receptor binding and this must be compensated for by additional mutations, creating a fitness barrier to accumulation of glycosylation sites. Positional conversion of glycosylation sites may represent another evolutionary feature of IAV that can, in part, circumvent the need to hyperglyosylate [14]. This term refers to the loss of a glycosylation site from one position that is accompanied by the appearance of another at a distinct site. Using *in silico* modeling, Sun *et al.* demonstrated positional conversion of glycans on the HA could occur for multiple reasons, including, to more effectively cover an antigenic site [123], however fitness costs associated with such changes must again be considered.

### 5.2. Balancing the Impact of HA Glycosylation on Innate and Adaptive Immunity

As discussed above, studies *in vitro* and in naïve mice have demonstrated that glycosylated viruses are sensitive to the innate immune activity of collectins and that both the number and position of glycans added to the HA can modulate recognition by collectins. In general terms, sequential addition of glycans to the head of HA increases sensitivity to SP-D [55,62], however specific sites appear to be particularly important in their ability to promote multivalent collectin binding. For H1N1, Asn_130/158 _[66], Asn_130/163_ [62] and Asn_95_ [66] (Asn_144/172_, Asn_144/177_ and Asn_104_, respectively) have been implicated in modulating sensitivity to SP-D whereas for H3N2 Asn_165_ [66,124] and Asn_246_ [63,125] (Asn_181_ and Asn_262_, respectively) are important sites, although for both subtypes additional sites have also been implicated [55,62,66]. 

In an immune setting, the number and location of glycans is also important in masking antigenic sites and in eliciting protective humoral responses against antigenically diverse virus strains. However, highly glycosylated IAV are recognized and inactivated by collectins of the innate immune system. As such, a fine balance must exist regarding the optimal degree and/or positioning of HA glycosylation to facilitate evasion of antibody-mediated neutralization while allowing adequate resistance to collectin-mediated defenses. Our recent studies demonstrated that addition of glycan at Asn_123_ (Asn_136_) to the HA of A(H1N1)pdm09 reduced its reactivity with polyclonal antisera raised in mice or ferrets [10]. Surprisingly, this glycan did not increase sensitivity to SP-D or alter virulence in naïve mice, suggesting that a complex glycan (which typically terminate in galactose or GalNAc and are therefore recognized less efficiently by SP-D [126]) occupied this site. Medina *et al.* also reported that addition of Asn_130_ (Asn_144_) to A(H1N1)pdm09 HA did not attenuate virulence in naïve mice but did play a major role in antigenic shielding and in increasing the breadth of the polyclonal antibody response elicited [32]. Clearly, limited recognition by innate immune collectins combined with effective masking of immunodominant epitopes on HA could be factors determining the optimal HA glycosylation pattern of emerging virus variants. 

**Figure 3:**
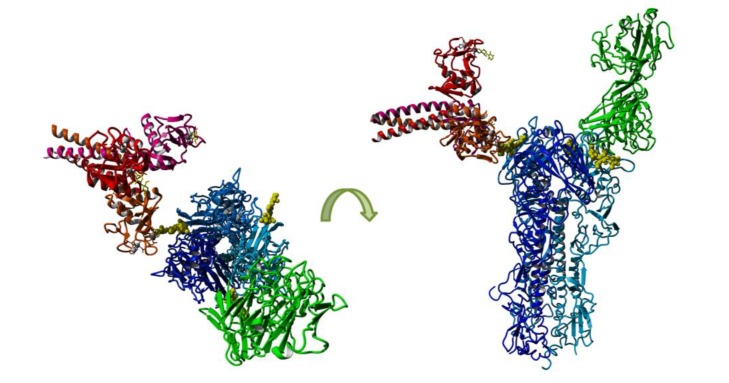
Schematic model showing recognition of glycan at Asn_165_ (Asn_181_) of H3 HA by SP-D or by Ab. An H3 trimer (blue) with glycosylation (yellow) at Asn_165_ (Asn_181_) is shown with one monomer in complex with an Ab (green) that is partially binding to glycan at Asn_165_ as seen in crystal structure PDB:1ken and another monomer in a modeled complex with a SP-D trimer (red, PDB:1pwb) binding the Asn_165_ glycan. Modeling and visualization were performed with Yasara [128].

For H3N2 IAV, oligomannose at Asn_165_ (Asn_181_) has been implicated as a major determinant of sensitivity to SP-D [66,124] and to modulate recognition of antigenic epitopes on the viral HA [43]. Recent studies used X-ray crystallography and molecular modeling to confirm that binding of SP-D to glycans at Asn_165_ (Asn_181_) on the HA of A/Aichi/1/1968 was likely to block the SIA binding site on HA, thereby contributing to antiviral activity [127]. Figure 3 models interactions between SP-D and antibody with H3 HA expressing glycan at Asn_165_ (Asn_181_). The orientation of antibody for epitope recognition is quite restricted as binding results from the cumulative effect of multiple specific interactions. In contrast, Ca^2+^-dependent interactions between the collectin CRD and glycan are predicted to be much more flexible and SP-D could be modeled to bind in several different orientations, including binding to multiple glycans on one HA or to glycans expressed on multiple HA trimers. Note that the glycan itself may also be quite flexible and therefore adopt different conformations relative to the HA trimer, adding further complexity to modeling such interactions. 

## 6. Conclusions

Oligosaccharides mask or modify antigenic sites on HA and their presence focuses variation on uncovered antigenic epitopes. While hyperglycosylation of HIV-1 gp160 is an effective strategy for deflecting neutralizing Abs the IAV HA does not appear capable of blocking all neutralization sites with oligosaccharides and maintaining its function. Indeed, while many studies report HA glycosylation to be associated with the evolutionary advantage of antigenic escape it is clear that this is often offset by reduced receptor affinity and/or other changes that affect virus fitness. Furthermore, as glycosylation enhances susceptibility of IAV to soluble and cell-associated lectins, the benefit provided to IAV via evasion of humoral immunity may be balanced by attenuation of infection due to enhanced innate immune recognition. In humans, optimal HA glycosylation patterns are likely to be the result of competing pressures associated with evasion of humoral immunity, maintenance of virus fitness and attenuation of pathogenicity following recognition by lectins of the innate immune system.
